# Disruption of GM2/GD2 synthase gene resulted in overt expression of 9-*O*-acetyl GD3 irrespective of Tis21^1^

**DOI:** 10.1111/j.1471-4159.2008.05232.x

**Published:** 2008-05

**Authors:** Keiko Furukawa, Wei Aixinjueluo, Takeshi Kasama, Yuki Ohkawa, Michiko Yoshihara, Yusuke Ohmi, Orie Tajima, Akio Suzumura, Daiji Kittaka, Koichi Furukawa

**Affiliations:** * Department of Biochemistry II, Nagoya University Graduate School of Medicine Nagoya, Japan; † Department of Immune System, Institute for Environmental Medicine Nagoya, Japan; ‡ Department of Biomedical Science, College of Life and Health Science, Chubu University, Kasugai Aichi, Japan; § Instrumental Analysis Research Center for Life Science, Tokyo Medical and Dental University Tokyo, Japan

**Keywords:** 9-*O*-acetyl GD3, brain, ganglioside, knockout, neuron, *O*-acetylation

## Abstract

GM2/GD2 synthase gene knockout mice lack all complex gangliosides, which are abundantly expressed in the nervous systems of vertebrates. In turn, they have increased precursor structures GM3 and GD3, probably replacing the roles of the depleted complex gangliosides. In this study, we found that 9-*O*-acetyl GD3 is also highly expressed as one of the major glycosphingolipids accumulating in the nervous tissues of the mutant mice. The identity of the novel component was confirmed by neuraminidase treatment, thin layer chromatography-immunostaining, two-dimensional thin layer chromatography with base treatment, and mass spectrometry. All candidate factors reported to be possible inducer of 9-*O*- acetylation, such as bitamine D binding protein, acetyl CoA transporter, or *O*-acetyl ganglioside synthase were not up-regulated. Tis21 which had been reported to be a 9-*O*-acetylation inducer was partially down-regulated in the null mutants, suggesting that Tis21 is not involved in the induction of 9-*O*-acetyl-GD3 and that accumulated high amount of GD3 might be the main factor for the dramatic increase of 9-*O*-acetyl GD3. The ability to acetylate exogenously added GD3 in the normal mouse astrocytes was examined, showing that the wild-type brain might be able to synthesize very low levels of 9-*O*-acetyl GD3. Increased 9-*O*-acetyl GD3, in addition to GM3 and GD3, may play an important role in the compensation for deleted complex gangliosides in the mutant mice.

*J. Neurochem.* (2008) **105,** 1057–1066.

GD3 ganglioside has been considered to be associated with cell proliferation and cell activation, as it is exclusively expressed in the fetal brain tissues at the proliferative stage of development (Yu *et al.* 1988). It can be induced in human T lymphocytes by stimulating with Concanavalin A or interleukin 2 (Yamashiro *et al.* 1995). It is also induced in fibroblasts introduced with oncogenic *ras* gene (Nakaishi *et al.* 1988). GD3 has been considered to be a melanoma-associated tumor antigen (Furukawa and Lloyd 1990), and been used as a target of antibody therapy in melanoma patients (Houghton *et al.* 1985). Recently, we demonstrated that GD3 expression actually enhanced the malignant properties of melanoma cells via increased tyrosine phosphorylation of adaptor molecules such as p130Cas or paxillin (Hamamura *et al.* 2005).

Besides GD3, a derivative of GD3 with *O*-acetylated sialic acid has been reported to be also associated with tumors. In particular, 9-*O*-acetyl GD3 was found in some of human melanomas (Cheresh *et al.* 1984) and in other tumor tissues (Kohla *et al.* 2002), suggesting that this structure is involved in the development and evolution of cancer (Schauer and Kamerling 1997; Angata and Varki 2002).

7(9)-*O*-acetyltransferase, the enzyme responsible for forming *O*-acetylated gangliosides, was demonstrated in bovine submandibular glands in 1970 (Schauer 1970). Recently, many attempts have been made to identify the gene without success, suggesting that a single gene product may not be able to catalyze the 9-*O*-acetylation of sialic acids. These attempts resulted in the identification of a putative acetyl-CoA transporter (named AT-1) cDNA (Kanamori *et al.* 1997) or of a cDNA for a novel transcriptional factor (or AGS, *O*-acetyl ganglioside synthase) (Ogura *et al.* 1996). Vitamin D-binding protein (vit-D bp) was also defined as a candidate for 9-*O*-acetylation-causing factor (Shi *et al.* 1988). Recently, Satake *et al.* identified Tis21 as an inducer of 9-*O*-acetylation of GD3 under the expression of GD3 (Satake *et al.* 2002). This gene was originally identified as a promoting factor of cell proliferation, and appears to also act as an inducing factor of the *O*-acetyltransferase.

In the mutant mice of GM2/GD2 synthase lacking all complex gangliosides, GM3 and GD3 accumulated at extremely high levels (Takamiya *et al.* 1996). Recently, we detected an additional band migrating between GM3 and GD3 in thin layer chromatography (TLC). This component could only be detected in the preparation without alkaline treatment, suggesting it is alkaline sensitive. Based on the sensitivity to neuraminidase treatment and alkaline treatment, TLC-immunostaining, and mass spectrometry, the band was identified as 9-*O*-acetyl GD3. Mechanisms for the neo-expression of 9-*O*-acetyl GD3 in the mutant mice were analyzed.

## Materials and methods

### Knockout (KO) mice

The generation of the KO mice of GM2/GD2 synthase (Takamiya *et al.* 1996) and GD3 synthase (Okada *et al.* 2002) was previously reported. Maintenance and genetic typing of these mice were performed according to the directions of the Ministry of Education, Culture, Sports, Science and Technology of Japan (MEXT). This study was approved by the Committee for Animal Experiment of Nagoya University Graduate School of Medicine.

### Extraction of glycolipids and TLC

Glycolipid extraction was performed as described previously (Furukawa *et al.* 1985). The brief and quick extraction was performed as previously described (Miyazaki *et al.* 1997), in which alkaline treatment step was skipped. Extracts with chloroform/methanol were directly applied to DEAE SephadexTM A-50 (Amersham Biosciences AB, Uppsala, Sweden) ion exchange column chromatography after desalting as described previously (Miyazaki *et al.* 1997). TLC was performed usually with chloroform/ methanol/0.2% CaCl_2_ (55 : 45 : 10), and bands were detected with resorcinol spray.

### Neuraminidase treatment of gangliosides

In order to clarify the core structures of individual bands in TLC, ganglioside mixtures were digested with a neuraminidase (*Clostridium perfringens*, Sigma, St Louis, MO, USA), and the products were desalted and analyzed in TLC/resorcinol spray.

### TLC-immunostaining

Using an anti-9-*O*-acetyl GD3 monoclonal antibody (mAb) Jones (Blum and Barnstable 1987) (kindly provided by Dr Colin Barnstable at Rockefeller Institute, New York) or mAb GMR2 (anti-9-*O*-acetyl GD3) (Seikagaku Kogyo, Tokyo), TLC-immunostaining was performed. After development of TLC, glycolipids were heat-blotted with an automated heat blotter (Atto, Tokyo) as described in the manufacturer's instruction, and soaked in 2% bovine serum albumin/PBS for the blocking at 4°C over night. The membrane was stained with the antibodies at 2–5 μg/mL for 2 h at 25°C, then detected with horseradish peroxidase-labeled anti-mouse IgG (H & L) antibody or anti-mouse μ antibody combined with Western Lightning™ chemiluminescence reagent (PerkinElmer LAS, Inc., Boston, MA, USA). TLC-immunostaining with mAb R24 (anti-GD3) was also performed. Detection of antibody binding was also performed with Immunostaining HRP-1000™ (Seikagaku Kogyo, Tokyo).

### Two-dimensional TLC

Two-D TLC was performed by changing the direction of TLC −90°. For the standard condition, an identical neutral solvent system was used to confirm the diagonal distribution of all components. Then, alkaline-sensitivity was analyzed by exposing TLC plates to NH_3_ vapor for 2 h in a sealed box between the first and the second development.

### Mass spectrometry

Mass spectrometry was performed to confirm the chemical structure of the new band, using the excised band from PVDF membrane on which gangliosides were transferred from an HP-TLC plate. Secondary ion mass spectrometry (SIMS) of the glycolipids was performed as described previously (Taki *et al.* 1995; Kasama *et al.* 1996). Negative ion mass spectra of glycolipids were recorded on a TSQ 700 quadrupole mass spectrometer (Thermo Fisher Scientific, Inc., Waltham, MA, USA) equipped with a cesium ion gun as follows. The glycolipid band on the PVDF membrane was excised (1.5 mm in diameter) and placed on a sample tip of mass spectrometer, and few microliters of triethanolamine (Wako Pure Chemical Industries, Osaka, Japan) was added as the SIMS matrix. The matrix with sample was bombarded with primary ion beam of Cs+ at 20 KeV. The ion multiplier was kept at 1.2 KV and the conversion dynode at 20 KV. The spectrum was accumulated several tens of scans.

### Reverse transcription-polymerase chain reaction

Expression levels of candidate genes for the inducing factor of *O*-acetylase were examined with total RNA from the nervous tissues of the wild-type (WT), GM2/GD2 synthase KO, and GD3 synthase KO mice. Total RNA 4 μg was served for reverse transcription in 50 μL mixture at 37°C for 90 min followed by 3 min incubation at 95°C. Usually, PCR was performed in 50 μL of reaction mixture using cDNA (2.5 μL), 2 mM dNTP, 25 mM MgCl_2_, Taq polymerase (0.25 μL), and 5 μM primers with a time course of 94°C for 3 min and 30 cycles of (94°C for 1 min, 55°C for 1 min, and 72°C for 1 min), then at 72°C for 7 min. Actually, PCR for AGS gene was performed at 60°C for annealing.

The products were analyzed by electrophoresis and ethidium bromide (EtBr) fluorescence under UV. Primer sequences used for PCR were as follows, vitD-bp: sense primer, 5′-CCAACCCTGAAAACCCTTAG-3′, anti-sense primer, 5′-CAGAGTGGTTCAGTTC AAGA-3′; AGS: sense primer, 5′-GCCTGGACGGCTCAGAGCAA-3′, antisense primer, 5′-TTGGGCTGGCAGGACTGAGC-3′; AT-1: sense primer, 5′-AACACTGTGTCTAATCTGGG-3′, antisense primer, 5′-CCAGTAATGTT GGCATCAGT-3′; Tis21: sense primer, AGTCTGAATGCTGCTACCAC-3′, antisense primer, 5′-CAGCCCTACAAGAATACCAG-3′; glyceraldehydes 3-phosphate dehydrogenase, (GAPDH): sense primer, 5′-GTCAGTGGTGGACCTGACCT-3′, antisense primer, 5′-TGCTGTAGCCAAATTCGTTG-3′.

### Uptake and expression of exogenous gangliosides by cultured astrocytes

Astrocytes from mouse brain were prepared as described previously (Mizuno *et al.* 2005). Astrocytes passaged more than three times were plated in microtiter plates (500 cells/well), and incubated over night. Then, GD3 was dried in a glass tube, and was resuspended in plain minimum essential medium with rigorous vortex. GD3 solution was added to the cells in the plates after washing twice with plain medium. Ganglioside expression was analyzed next day by an immunofluorescence assay. Serially diluted antibodies with PBS containing 3% fetal calf serum was added to the cells, and incubated for 1 h at 25°C. After washing twice, FITC-conjugated second antibodies (goat anti-mouse IgG or anti-mouse μ chain) were added and incubated for 30 min. After washing, cells were examined under a fluorescence microscopy.

## Results

### TLC of WT and KO mice brain gangliosides

**Fig. 1:**
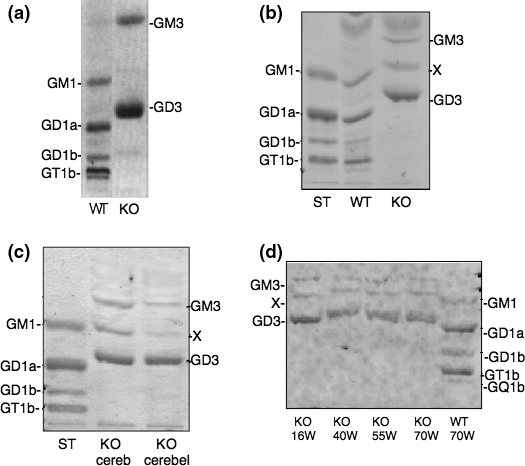
A new band in ganglioside fractions from the mutant mice lacking complex gangliosides. (a) TLC of brain gangliosides from the WT and KO mice prepared via alkaline treatment. (b) TLC of brain gangliosides from the WT and KO mice prepared by non-alkaline condition. (c) Ganglioside fractions from cerebrum and cerebellum tissues of a KO mouse. Ten mg tissue-derived gangliosides were applied for all samples. (d) Ganglioside fractions of the KO brains at different ages were compared in TLC/resorcinol. The new band was indicated by X in b–d.

Ganglioside fractions were extracted as previously reported (Furukawa *et al.* 1985). Then, gangliosides were also prepared with a brief method without alkaline treatment, where acidic fractions were directly isolated from chloroform/methanol extracts using DEAE SephadexTM A-50 ion-exchange column (Miyazaki *et al.* 1997). In the standard separation, only GM3 and GD3 could be found in the extracts from the mutant mice (Takamiya *et al.* 1996) (Fig. 1a). In contrast, the brief preparation resulted in the appearance of a new band between GM3 and GD3, showing similar band intensity to that of GM3 (Fig. 1b). This TLC pattern was essentially same in cerebrum and cerebellum (Fig. 1c). The new band was present persistently from 16 to 70 weeks after birth (Fig. 1d).

### Neuraminidase treatment

**Fig. 2:**
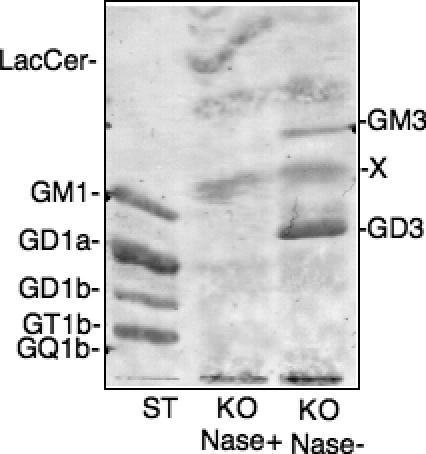
Neuraminidase treatment of gangliosides from the KO mouse brain. Gangliosides were incubated in the presence or absence of neuraminidase and the products were analyzed in TLC after desalting. ST, bovine brain gangliosides. TLC was developed with a solvent system of chloroform: methanol: 0.2% CaCl_2_ (55 : 45 : 10). Bands were visualized by resorcinol spray. The new band was indicated by X.

In order to clarify the structures of the bands in TLC, ganglioside mixtures were digested with a neuraminidase, and the products were analyzed in TLC. Gangliosides from WT mice brain became a single band at the migration level of GM1 (data not shown), corresponding to the previous reports (Yamamoto *et al.* 1995). On the other hand, no GM1-like band could be found in the null mutants. In stead, a definite band was found at just above GM1 site, showing identical migration behavior to the original migration site of the novel band (Fig. 2). This result suggested that the unknown component was neuraminidase-resistant. An intense band above GM3 was newly found in the KO extracts after the neuraminidase treatment (Fig. 2). This brown band distinct from violet bands with resorcinol was found only in the KO sample, suggesting the product was a neutral (non-sialylated) glycolipid, lactosylceramide derived from GM3 and GD3.

### TLC-immunostaining

**Fig. 3:**
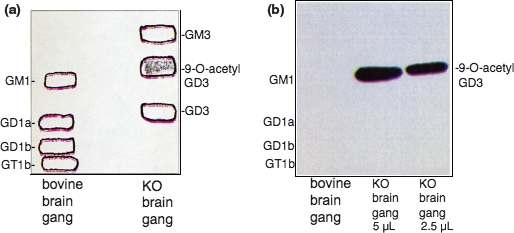
The new component was 9-*O*-acetyl GD3 as analyzed by TLC-immunostaining. (a and b) TLC-immunostaining of ganglioside fractions from a KO mouse with mAb Jones. (a) TLC pattern of gangliosides from a KO mouse as well as bovine brain to show the migration sites of bands. After TLC, primulin-stained bands were marked with a pencil, then transferred to PVDF by heat-blotting. (b) After blotting, bands were stained with mAb Jones (ascites, 1 : 100 dilution) and a chemiluminescence detection system.

To identify the new band, TLC-immunostaining using mAb Jones was performed. An intensely stained band was found at the same migration site as the new band indicated by X in Fig. 3a and 3b. This result indicated that the unknown band was 9-*O*-acetyl-GD3.

### Comparison of TLC-immunostaining pattern between the wild-type and the KO mice

**Fig. 4:**
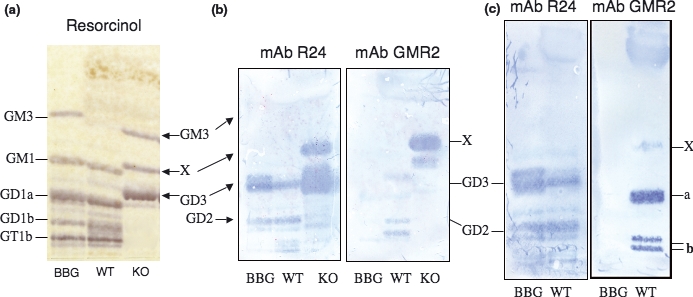
The new component was detectable even in the WT mouse brain extracts. To clarify whether 9-*O*-acetyl-GD3 was expressed in the WT mouse brain, TLC-immunostaining was rigorously performed. (a) Resorcinol pattern of the acidic fractions from WT and KO mice. BBG is a standard (bovine brain gangliosides). (b) TLC-immunostaining of the samples as shown in (a) with mAb R24 and mAb GMR2. mR24 was used at 1 : 100 dilution of ascites, and mAb GMR2 was at ×10 dilution of the commercial product (1 μg/mL). (c) Twice amounts of gangliosides were applied, and incubated with the substrate at the final step for longer time, leading to the detection of a faint band at the site of X. TLC-immunostaining with mAb Jones also showed same results.

To clarify whether the wild-type mice express some levels of 9-*O*-acetyl-GD3, further TLC-immunostaining was performed. Using acidic fractions from WT and KO mouse brains as shown in Fig. 4(a), TLC-immunostaining was performed with anti-GD3 mAb R24 and anti-9-*O*-acetyl GD3 mAb GMR2. mAb R24 stained GD3 in bovine brain gangliosides (moderately), WT mouse brain extracts (weakly), and in the KO mice brain (strongly) (Fig. 4(b)). As expected, mAb GMR2 stained a strong band of probably 9-*O*-acetyl-GD3 at the site of X. No corresponding band was detectable in the WT sample. When, double amounts of gangliosides were stained with longer incubation with the substrate, a very faint band appeared at the migration site of X, suggesting that very low amount of 9-*O*-acetyl-GD3 was present in the brain of the WT mice (Fig. 4(c)). Additional bands (indicated as a and b) were observed under this condition, whereas the identity of them was not known.

### 2D TLC

**Fig. 5:**
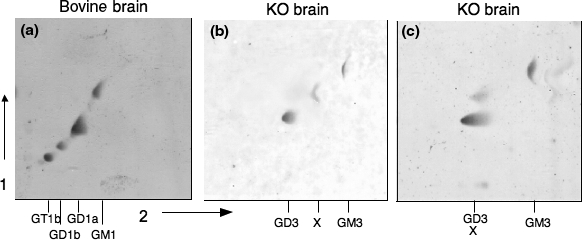
The new component was 9-*O*-acetyl GD3 as analyzed by 2D TLC. 2D TLC of gangliosides extracted from a KO mouse brain showing an alkaline-labile component. (a) Gangliosides from bovine brain as a standard. Gangliosides from a WT mouse showed a similar pattern (data not shown). (b and c) Gangliosides from a KO mouse. Alkaline treatment of the plate was done between the 1st and the 2nd development in (c), but not in (b). For all plates, the 1st and the 2nd developments were done with the identical solvent system of chloroform : methanol: 0.2% CaCl_2_ (55 : 45 : 10). Resorcinol spray was used for the spot detection.

In order to further investigate the nature of the new band in the null mutants, we examined the 2D TLC pattern of gangliosides from WT and KO mice brains. After TLC of gangliosides using C: M: 0.2% CaCl_2_ (55 : 45 : 10), the TLC plates were exposed to NH_3_ vapor for 2 h. Then, TLC was developed by turning the direction −90° using the same neutral solvent. As expected, all ganglioside components were aligned on the diagonal line in WT gangliosides as well as bovine brain gangliosides (Fig. 5a), indicating all these spots consisted of alkaline-resistant structures. When gangliosides from KO mice were developed with a neutral solvent system in both first and second dimension, three spots were found on the diagonal (Fig. 5b). On the other hand, KO mice samples showed a non-diagonal pattern, i.e., a new component migrated more slowly in the second chromatography when the plate was exposed to NH_3_ after the 1st dimension (Fig. 5c), suggesting this band is alkaline sensitive and was converted to GD3.

### Mass spectrometry

**Fig. 6:**
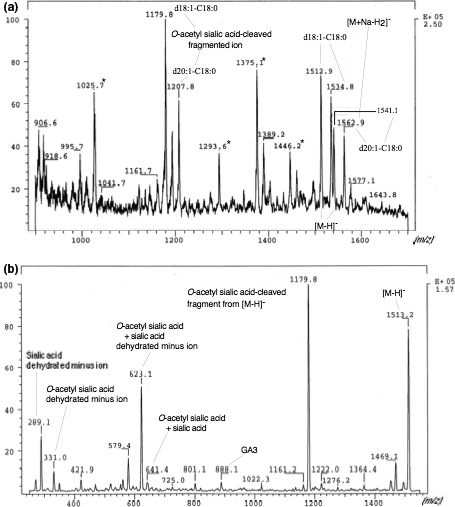
Mass spectrometry to confirm the presence of *O*-acetylation in GD3. (a) Gangliosides from a KO mouse brain was separated in TLC and visualized with primulin. The new component was cleaved from PVDF membrane on which gangliosides were transferred and secondary ion mass spectrometry was performed as described in the Experimental Procedures. Presence of *O*-acetylated gangliosides containing d20:1-C18:0 and d18:1-C18:0 were suggested. (b) MS/MS spectrum measured from *m*/*z* 1513.2 ([M-H]^−^) as a precursor was shown. Peaks derived from precursor ions were indicated in the figure. These results indicated that the band contained *O*-acetyl GD3, though the accurate acetylated site could not be determined.

Mass spectrometry was performed using the novel band from PVDF membrane on which gangliosides were transferred from an HP-TLC plate. As shown in Fig. 6a, presence of *O*-acetylated gangliosides containing d20:1-C18:0 and d18:1-C18:0 were suggested. In A, *m*/*z* 1534.8 and 1562.9 were [M+Na-H_2_]^−^. *m*/*z* 1512.9 and *m*/*z* 1541.1 were [M-H]^−^. *m*/*z* 1179.8 and 1207.8 were of fragment ions from which *O*-acetyl sialic acid was cleaved. Based on ceramide structures, *m*/*z* 1179.8, 1512.9, 1534.8 were from gangliosides with d18:1-C18:0, and *m*/*z* 1207.8, 1541.1, 1562.9 were from gangliosides with d20:1-C18:0. In MS/MS spectrum of *m*/*z* 1513.2 ([M-H]^−^) (Fig. 6b), *m*/*z* 1179.8 was a fragment ion derived from [M-H]^−^ from which *O*-acetyl sialic acid was cleaved. *m*/*z* 289.1 (correct *m*/*z* 290) corresponded to sialic acids (dehydrated minus ion). *m*/*z* 331.0 (correct *m*/*z* 332) corresponded to *O*-acetyl sialic acid (dehydrated minus ion). *m*/*z* 623.1 corresponded to *O*-acetyl sialic acid + sialic acid (dehydrated minus ion). *m*/*z* 641.1 corresponded to *O*-acetyl sialic acid + sialic acid. *m*/*z* 888.1 corresponded to the ion of GA3 structure. These results indicated the band contained *O*-acetyl GD3, though the exact acetylated site could not be determined.

### Expression levels of candidate genes for the inducing factor of *O*-acetylase

**Fig. 7:**
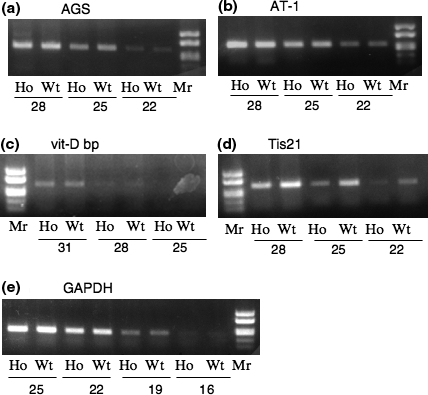
Expression levels of genes possibly involved in the expression of 9-*O*-acetyl GD3. Results of RT-PCR of AGS (a), AT-1 (b), vit-D bp (c) and Tis21 (d) genes. A result of GAPDH gene was shown as a control (e). Products of RT-PCR with cycles indicated in each panel using mRNA from KO and WT mice were separated in an acrylamide gel, and EtBr-stained bands were obtained, showing almost equivalent levels (AGS, AT-1, vit-D bp as well as GAPDH) or reduced expression levels in KO (Tis21).

**Fig. 8:**
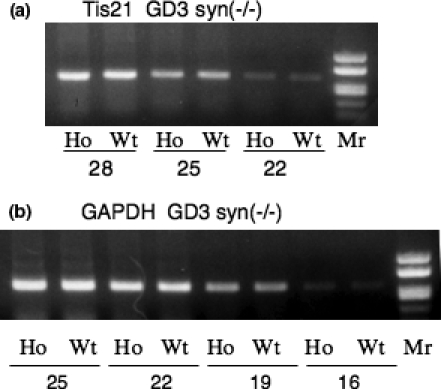
Expression levels of Tis21 gene in GD3 synthase KO mice. (a) RT-PCR was performed to analyze Tis21 gene expression in GD3 synthase KO mice as well as the Wt mice as described above. (b) Expression levels of GAPDH gene as a control with cycles indicated in the figure. Almost equivalent levels of Tis21 mRNA in the presence/absence of GD3 were shown.

In order to clarify that involvement of several genes that were previously reported to be associated with *O*-acetylation of GD3 in the neo-expression of 9-*O*-acetyl GD3 in the mutant mice, RT-PCR was performed. None of the genes examined, i.e., Tis21, vitD-bp, AT-1, and AGS, showed up-regulation in GM2/GD2 synthase gene KO mice compared to the WT mice (Fig. 7). In particular, the expression level of Tis21 that had been reported to be expressed under GD3 expression rather decreased in the KO brain (Fig. 7d). Since it has been suggested that Tis21 was induced by GD3 expression leading to the induction of machinery for 9-*O*-acetylation, the mRNA expression levels in GD3 synthase KO mice were also examined. However, no definite changes in the expression levels of Tis21 could be observed (Fig. 8).

### Uptake and acetylation of GD3 in astrocytes

**Fig. 9:**
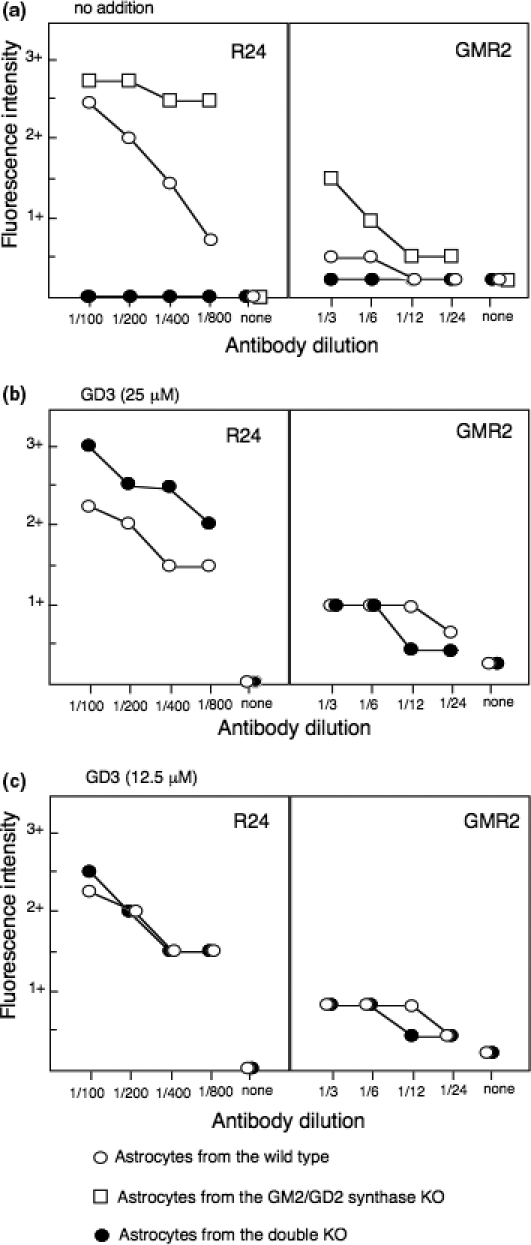
Expression of GD3 and 9-*O*-acetyl-GD3 after addition of exogenous GD3 to cultured astrocytes. Astrocytes were cultured with or without GD3 for 24 h, and expression of the gangliosides were analyzed by immunofluorescence assay as described in the materials and methods. The intensity of the stained fluorescence was scored as follows, 3+, bright; 2+, moderate; 1+, weak; ±, marginal. Open squire represents cells from the KO (GM2/GD2 synthase) mouse. Open circle represents those of the WT mouse, and closed ones were of the double KO (GM2/GD2 synthase and GD3 synthase).

**Fig. 10:**
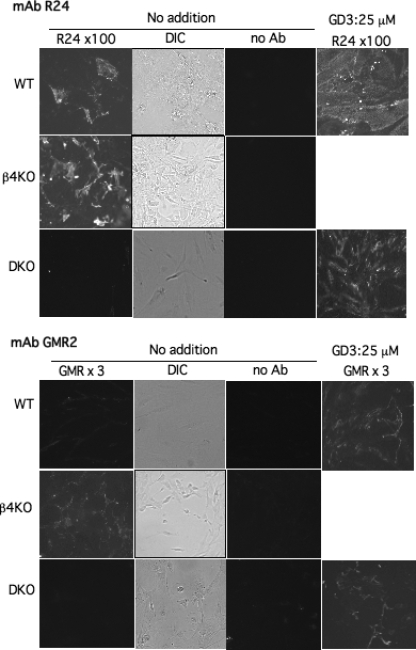
Pictures of the immunofluorescence assay as shown in Fig. 9. Pictures were taken with identical conditions, i.e., fluorescence samples were at 3 s of the exposure, and DIC was at 1 s exposure. Results of mAb R24 (1 : 100 dilution) and mAb GMR2 (1 : 3 dilution) were shown. Since the KO (β4KO) already showed positive reaction for 9-*O*-acetyl-GD3 before the GD3 treatment, we did not try to add GD3 (they had extremely high amounts of GD3).

In order to clarify whether WT or other mutant mouse brain had enzyme activity to synthesize 9-*O*-acetyl-GD3, expression of 9-*O*-acetyl-GD3 as well as GD3 was analyzed after addition of GD3 to the culture medium of astrocytes. When nothing was added to the medium, strong expression of GD3 in the astrocytes from the KO mice were observed (Fig. 9(a)). WT astrocytes also expressed GD3 at a fairly level. As expected, astrocytes from the double KO mice (GM2/GD2 synthase and GD3 synthase) showed no GD3. As for 9-*O*-acetyl-GD3, the KO-derived astrocytes showed a low, but definite level of 9-*O*-acetyl-GD3 as detected by mAb GMR2. WT samples showed marginal expression. When exogenous GD3 (25 μM) was added, high levels of GD3 was observed in both the double KO- and the WT-derived astrocytes (Fig. 9(b)). Low levels of 9-*O*-acetyl-GD3 were also found in both types of mice. Addition of half amount of GD3 resulted in essentially same effects with slightly less intensity (Fig. 9(c)). In Fig. 10, pictures of cells observed under fluorescence microscopy were shown.

## Discussion

9-*O*-acetyl GD3 has been considered to be associated with tumors. Thus, 9-*O*-acetyl GD3 was found in some of human melanomas (Cheresh *et al.* 1984; Ritter *et al.* 1990) and basal cell sarcomas (Fahr and Schauer 2001), suggesting this structure is involved in cancer evolution (Schauer and Kamerling 1997;Angata and Varki 2002), though no direct evidence has been shown. On the otherhand, 9-*O*-acetylation in influenza C protein transgenic mice resulted in the arrest of cell division at the early stage of development (Varki *et al.* 1991).

7(9)-*O*-acetyltransferase, the enzyme responsible for the acetylation of gangliosides was first detected in bovine submandibular glands (Schauer 1970). Recently, many trials have been performed to identify the gene mainly by expression cloning system, resulting in failure. These results suggest that a single gene product may not be able to catalyze the reaction of 9-*O*-acetylation onto sialic acids or sialic acid-nucleotides. Thus, the regulatory mechanisms for the synthesis of *O*-acetylated sialic acids is not well understood because of the unavailability of the cDNA of the enzyme coding 9-*O*-acetylase. The active form may exist as a molecular complex of several components.

In the mutant mice of GM2/GD2 synthase gene, 9-*O*-acetyl GD3 is expressed at an extremely high level. In the early reports, gangliosides were isolated via alkaline treatment, resulting in the elimination of *O*-acetyl compounds (Fig. 1a) (Takamiya *et al.* 1996). Here, we demonstrated the identity of the new band using multiple approaches. First of all, the component was sialidase-resistant, while two major bands were completely digested and converted to lactosylceramide. Second, the band was specifically detected by an anti-9-*O*-acetyl GD3 mAb in TLC-immunostaining. Third, the component showed altered migration in TLC after alkaline treatment, resulting in the identical migration with that of GD3. Fourth, mass spectrometry of the component revealed that it contained *O*-acetyl base in GD3. Taken together, the unknown band found in the brain tissues from the mutant mice turned out to be 9-*O*-acetyl GD3.

Mechanisms for the neo-synthesis of 9-*O*-acetyl GD3 in the mutant mice were analyzed by examining the changes in the expression levels of mRNAs for genes that had been reported to be associated with 9-*O*-acetyl GD3. Rat AGS (Ogura *et al.* 1996) containing high homology with mouse fat globule membrane protein was cloned by expression cloning using GD3 synthase-transfected CHO cells, and was considered as a putative *O*-acetyltransferase. This gene was reported to be up-regulated in stellate cells during liver fibrogenesis (Lee *et al.* 2003). AT-1 was also isolated by expression cloning as a membrane protein required for the formation of *O*-acetylated gangliosides, and concluded to be a putative acetyl-CoA transporter gene (Kanamori *et al.* 1997). Shi *et al.* also isolated cDNA clones by expression cloning using CHE-FcD as a detection probe, and identified that one of them was vit-D bp (Shi *et al.* 1988). This could induce 9-*O*-acetylation on COS cells expressing α2,6-linked sialic acids. As these authors pointed out, it is unlikely that the products of all these genes act as an *O*-acetyltransferase. Recently, Satake *et al.* reported that GD3-specific 9-*O*-acetyltransferase was induced by Tis21, expression of which was enhanced by the transfection of GD3 synthase cDNA into CHO cells (Satake *et al.* 2002). They concluded that GD3 expression triggered Tis21 expression. This hypothesis was confirmed here in the mutant mice exhibiting accumulation of GD3 as well as GM3, and this seemed not the case in the mutant mice. In the RT-PCR, none of mRNAs examined showed significant up-regulation. Surprisingly, Tis21 showed rather a reduced level in the mutant mice, suggesting that high expression of 9-*O*-acetyl GD3 suppressed the Tis21 expression probably as a feed back inhibition.

Accumulation of the precursor GD3 might be a crucial factor in the enhanced synthesis of 9-*O*-acetyl GD3, and the synthetic activity itself (9-*O*-acetylransferase) might not be changed, although no definite evidence could be shown. The results of TLC-immunostaining with WT mouse brain extracts suggested that even WT mice could synthesize 9-*O*-acetyl-GD3, though at an extremely low level. Martin *et al.* reported that specific sialyltransferases influence the *in vivo* regulation of *O*-acetylation, i.e., loss of *O*-acetylation in ST6GalI and ST3GalI null mice (Martin *et al.* 2002). This seems quite reasonable, because these enzymes are indispensable to synthesize the precursors or acceptors of the *O*-acetylation. By analogy, accumulation of GD3, the precursor of 9-*O*-acetyl GD3, could lead to the neo-synthesis of 9-*O*-acetyl GD3 based on its high level in the mutant mice of GM2/GD2 synthase.

Since over-expression of GD3 might have induced Tis21 gene expression, leading to the induction of 9-*O*-acetylation of GD3 (Satake *et al.* 2002), Tis21 expression in GD3 synthase KO mice was examined to clarify whether GD3 is essential for the induction of Tis21. Contrary to expectation, Tis21 mRNA levels were not altered in the GD3 synthase KO mice, indicating that Tis21 mRNA was not affected even in the absence of GD3. Exogenously added GD3 was reported to induce *O*-acetyltransferase in CHO cells (Chen *et al.* 2006). Whether neo-expression of 9-*O*-acetyl GD3 is really dependent on the neo-expression of an *O*-acetyltransferase because of GD3 or the accumulation of GD3 merely prompts the synthesis of 9-*O*-acetyl GD3 remains to be investigated. It seems more likely that *O*-acetylase was induced directly or indirectly by the high levels of GD3 regardless of Tis21, or the existing level of *O*-acetylase was sufficient to increase 9-*O*-acetyl GD3 levels in the presence of huge amount of GD3. At least we can conclude that Tis21 is probably not an inducing factor of such an *O*-acetyltransferase in GM2/GD2 synthase KO mice.

As Chen reported (*Chen et al.* 2006), it might be possible that accumulated GD3 induced synthetic machinery of 9-*O*-acetyl-GD3 in the KO mice. As shown in Fig. 9 and 10 together with the results in Fig. 4, low levels of 9-*O*-acetyl-GD3 were already found in the WT mouse brain. Therefore, it seems reasonable that the expression of 9-*O*-acetyl-GD3 in the KO mouse brain became overt because of the increased synthesis based on the increased precursor structure.

Although 9-*O*-acetyl GD3 or 9-*O*-acetylated sialic acid-containing mucins have been considered to be involved in the malignant features of tumor cells, their roles in cell proliferation and invasion have not been clearly demonstrated. A significant role for the *O*-acetylation of GD3 in the induction of apoptosis has also been reported (Malisan *et al.* 2002). However, it is not clear whether modulation of GD3 is important or 9-*O*-acetyl sialic acid itself has a unique function. The mutant mice lacking complex gangliosides showed much milder phenotypes than expected (Takamiya *et al.* 1996). Accumulation of GM3 and GD3 was considered to compensate the functions of the lost structures, and to attenuate the morphological and functional defects because of the lack of the complex gangliosides. Neo-synthesis of 9-*O*-acetyl GD3 might also play an important role in the attenuation of the abnormal phenotypes of GM2/GD2 synthase KO mice. In fact, 9-*O*-acetyl GD3 was appeared to participate in neuronophilic as well as gliophilic migration of subventricular zone explants (Miyakoshi *et al.* 2001), and granule cell migration in the developing cerebellum (Santiago *et al.* 2001), suggesting its roles in the protection of neuronal death or reconstruction of the degenerated nervous system in the KO mice (Sugiura *et al.* 2005). In order to investigate these mechanisms, it seems critical to define the enzyme and other essential molecules responsible for the *O*-acetylation of sialic acids.

## Abbreviations used

2D TLC: two-dimensional TLC
AGS: *O*-acetyl ganglioside synthase, GAPDH, glyceraldehydes 3-phosphate dehydrogenase
KO: knockout
OAG: *O*-acetyl ganglioside
RT-PCR: reverse transcription-polymerase chain reaction
SIMS: secondary ion mass spectrometry
TLC: thin layer chromatography
vit-D bp: vitamin D binding protein
WT: wild-type
